# Racial Disparities and Personal Responsibility Incentives in Medicaid

**DOI:** 10.1111/1475-6773.70139

**Published:** 2026-06-10

**Authors:** David M. Craig, Elaine M. Hernandez, Elizabeth M. Anderson, Yvette H. Tran, Erik S. Parker, Justin Blackburn, Sumedha Gupta

**Affiliations:** ^1^ Department of Interdisciplinary Studies Indiana University Indianapolis School of Liberal Arts Indianapolis, Indiana USA; ^2^ Department of Sociology Indiana University Bloomington Bloomington, Indiana USA; ^3^ Department of Sociology University of Kansas College of Liberal Arts and Sciences Lawrence, Kansas USA; ^4^ Department of Health Policy and Management Indiana University Indianapolis Richard M. Fairbanks School of Public Health Indianapolis, Indiana USA; ^5^ Department of Epidemiology and Biostatistics School of Public Health‐Bloomington, Indiana University Bloomington Bloomington, Indiana USA; ^6^ Department of Economics Indiana University Indianapolis Indianapolis, Indiana USA

**Keywords:** cost‐sharing, health insurance enrollment, Medicaid, personal responsibility, policy evaluation, racial disparity

## Abstract

**Objective:**

To determine whether personal responsibility incentives in Medicaid differentially affect enrollment and the comprehensiveness of plan benefits among members who are non‐Hispanic Black and non‐Hispanic White.

**Study Setting and Design:**

We conducted an interrupted time series analysis to estimate trends in racial disparity ratios of enrollment across more comprehensive Healthy Indiana Plans (HIP) before and during the COVID‐19 Public Health Emergency (PHE) when the state suspended personal responsibility incentives, including monthly premium contributions.

**Data Sources and Analytic Sample:**

We analyzed restricted‐access administrative data from the Indiana Family and Social Services Administration from 2018 through 2023. The analytic cohort comprised 939,667 non‐Hispanic Black and non‐Hispanic White adults (19–64 years) enrolled in one of four HIP tiers, including HIP Plus or HIP Basic, and HIP State Plan Plus or HIP State Plan Basic in which presence of a qualifying health condition is required for eligibility.

**Principal Findings:**

Before the PHE, members who are non‐Hispanic Black were approximately 23 percentage points less likely to be in the more comprehensive HIP Plus plan relative to members who are non‐Hispanic White. An increase in the disparity ratio of 0.076 points toward parity (95% CI, 0.054–0.097 points) for HIP Plus recipients was observed following suspension of personal responsibility incentives during the PHE. After an administrative upgrade of all HIP Basic recipients to HIP Plus plans during July 2021, this disparity ratio increased an additional 0.146 points from the start of the PHE (95% CI, 0.141–0.151 points) to 0.994 (95% CI, 0.993–0.994).

**Conclusions:**

Personal responsibility incentives in Medicaid are associated with substantial and persistent racial disparities in enrollment and plan comprehensiveness. The study indicates that while the temporary removal of these incentives can reduce disparities, proactive policy interventions may be necessary to achieve and maintain equitable access to care.

## Lay Summary

- What is known on this topic?
  ○ State Medicaid programs have been permitted to enact personal responsibility provisions such as requiring premium, copayment, health savings account contributions, or participation in job‐seeking or community engagement activities.
  ○ Failure to meet personal responsibility requirements can lead to administrative disenrollment or reduced benefits, but can incentivize enrollees to seek employment and use preventive care.
  ○ Certain types of personal responsibility provisions contribute to racial and ethnic disparities in Medicaid coverage and access to recommended health care.
- What this study found?
  ○ In Indiana, temporary suspension of personal responsibility provisions was followed by a reduction in racial enrollment disparity by a statistically significant 8 percentage points in the more comprehensive plan.
  ○ An additional 15 percentage points of racial enrollment disparity were reduced following state administrative action to upgrade members remaining on a limited‐benefit plan.
  ○ Temporary suspension of Medicaid redetermination and personal responsibility requirements during the PHE did not entirely alleviate racial disparities in enrollment and plan comprehensiveness in Indiana.

## 1 Introduction

Following the passage of the Patient Protection and Affordable Care Act (ACA, P.L. 111–148, as amended), seven states expanded the income eligibility threshold for adults to qualify for Medicaid coverage using Section 1115 waivers. These waivers included “personal responsibility” provisions such as the requirements for enrollees to pay premiums or copayments, make health savings contributions, and participate in seeking employment or community engagement. In states with these provisions, failure to meet the requirements can lead to administrative disenrollment or reduced benefits. Collectively, experts have commented that the consequences of personal responsibility provisions may either (1) complicate the process of gaining and maintaining coverage and thus lead to negative results, particularly for enrollees in poor health, or (2) may incentivize enrollee engagement to seek preventive care and/or employment [1, 2, 3]. Furthermore, evidence suggests cost‐sharing requirements (e.g., premiums and copayments) in Medicaid may result in racial and ethnic disparities in participation in coverage and access to recommended healthcare [4, 5, 6, 7, 8, 9]. However, empirical evidence is limited for evaluating whether the interaction of personal responsibility provisions affects enrollment and continuous coverage differentially by race in Medicaid.

This study focuses on the Healthy Indiana Plan (HIP), which became Indiana's Medicaid Expansion plan in 2015 via a Section 1115 waiver. Modeled on consumer‐driven health plans, the original HIP was launched in 2008 to cover roughly 40,000 low‐income adults. This pioneering Medicaid health plan added vision and dental coverage and wellness rewards to standard medical coverage for those members who navigated personal responsibility incentives [10]. This original incentive structure was maintained in the Section 1115 waiver to expand eligibility in 2015 with the policy goal of empowering members “to take charge of their own health care needs.” [11] Subsequent enrollment grew tenfold to around 400,000 low‐income adults in Indiana. During the COVID‐19 Public Health Emergency (PHE), HIP enrollment exceeded 800,000 members [12]. HIP's enrollment growth and comprehensive benefit options have enhanced healthcare coverage for low‐income adults in Indiana, although its combination of personal responsibility incentives resulted in fewer gains prior to the pandemic than neighboring states with Medicaid waivers [13].

HIP is designed as a multi‐tiered benefits plan with personal responsibility incentives to maintain or regain coverage in more comprehensive benefits. Newly enrolled adults start in the more comprehensive tier (i.e., HIP Plus) and remain in this tier if they pay monthly income‐based premiums ranging between $1 and $20 into the Personal Wellness and Responsibility (POWER) Account, which is similar to a health savings account, plus an additional 50% surcharge for tobacco use [14]. Failure to pay triggers an administrative downgrade of those recipients earning between 0–100% of the Federal Poverty Level (FPL) into HIP Basic or a termination of coverage for recipients with incomes between 101–138% FPL. HIP Basic excludes vision and dental coverage and imposes copayments for each healthcare visit, medication, and inpatient stay. Recipients with complex medical conditions are reassigned to separate HIP State Plus and HIP State Basic plans, which have the same premium (Plus) versus copayment (Basic) requirements. Both State plans, however, have the full benefits of HIP Plus as well as enhanced benefits such as transportation [15].

The 2020 Families First Coronavirus Response Act increased federal Medicaid funding contingent upon states suspending eligibility redeterminations during the PHE, allowing Medicaid enrollees to retain coverage. Nationwide, Medicaid enrollment increased by nearly 23 million from March 2020 to March 2023 [16]. After Congress lifted the “suspension” on March 31, 2023, states resumed redeterminations (i.e., the Medicaid “unwinding”). Between March 2023 and April 2024, 18.7 million people were disenrolled from Medicaid coverage as a result of being no longer eligible or for procedural reasons [17].

From February 2020 to March 2023, the adult Medicaid population grew faster in Indiana (70%) than in the 32 other states that expanded Medicaid prior to January 2020 [18]. This increase was concentrated in HIP plans, which grew by 75% by June 2022 [19]. Among states with waivers to charge premiums in their Medicaid Expansion plans, only Indiana requires premiums from members starting at 0% FPL. Other states start their premiums at 50% FPL or at 100% FPL [20]. In conjunction with pausing redeterminations, Indiana suspended all Medicaid cost‐sharing (i.e., premiums and copayments) during the PHE, offering a valuable opportunity to explore how reducing administrative burdens, including personal responsibility incentives, can affect health coverage inequities. We hypothesize that if personal responsibility provisions disproportionately affect non‐Hispanic Black recipients, the absence of administrative downgrades and coverage terminations will be associated with enrollment parity with non‐Hispanic White recipients. At the time of this study, cost‐sharing remained suspended in HIP while its current 2020 waiver underwent court‐ordered review by the Centers for Medicare and Medicaid Services [21].

## 2 Methods

**FIGURE 1:**
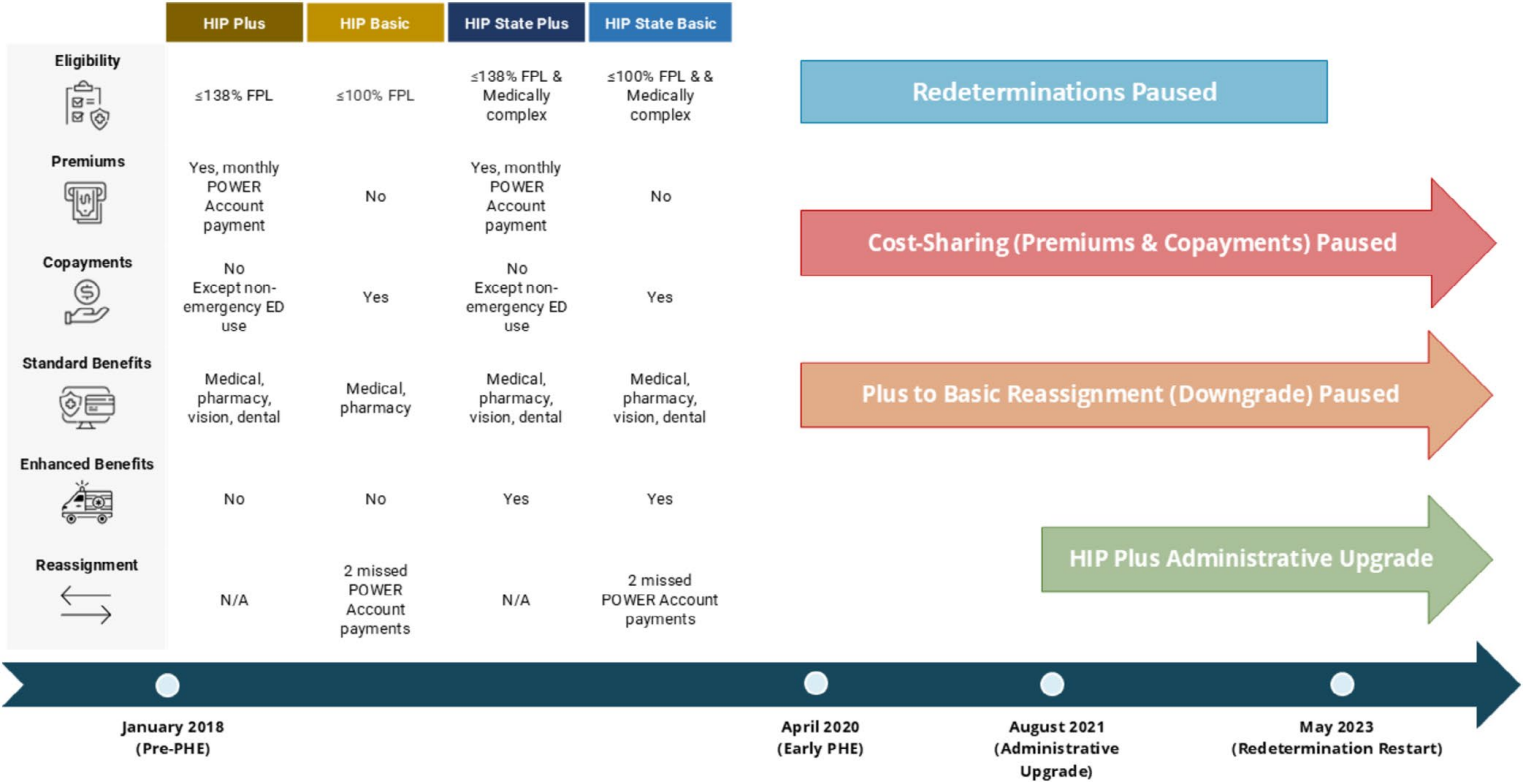
Healthy Indiana Plan Tiers and Rule Changes during the COVID‐19 Public Health Emergency. Description of Healthy Indiana Plan Tiers and Rule Changes during the COVID‐19 Public Health Emergency (PHE). This figure summarizes key features of Healthy Indiana Plan (HIP) tiers by eligibility, premiums, copayments, standard benefits, enhanced benefits, and reassignment rules. HIP consists of four main tiers varying in cost‐sharing responsibilities and comprehensiveness of benefits: HIP Plus, HIP Basic, HIP State Plus, and HIP State Basic. Enrollees with medical complexity qualify for HIP State Plus or State Basic Plans, which provide enhanced benefits such as non‐emergency transportation. Prior to the PHE period, HIP Plus and HIP State Plus enrollees pay an income‐based monthly premium into an account similar to a health savings account (POWER account payment) ranging between $1 and $20, with tobacco users facing a 50% surcharge. HIP Plus and HIP State Plus enrollees with two consecutive missed POWER account payments and incomes less than or equal to 100% of the federal poverty level (FPL) are downgraded (reassigned) to HIP Basic and HIP State Basic, respectively, but can opt to re‐enroll in HIP Plus or HIP State Plus at their redetermination date or remain in Basic tiers. HIP Plus enrollees with two consecutive missed POWER Account payments and incomes above 100% FPL are disenrolled. Once downgraded into HIP Basic, enrollees are subject to a limited benefits plan. Both HIP Basic and HIP State Basic enrollees must pay copayments, which vary by service type: Physician visit ($4), monthly prescription ($4–8), and inpatient stay ($75). At the start of the PHE (April 2020), Indiana paused redeterminations, cost‐sharing requirements, and downgrading of HIP Plus and HIP State Plus enrollees into Basic plans. In August 2021, Indiana administratively upgraded HIP Basic enrollees into HIP Plus. HIP State Basic enrollees were not upgraded because once cost‐sharing requirements were paused, HIP State Plus and HIP State Basic were identical. Indiana restarted redeterminations May 2023; however, cost‐sharing remains suspended pending litigation. Consequently, Indiana has not collected POWER Account payments and copayments since April 2020. Thus, HIP Plus and HIP State enrollees with missed POWER Account payments have not been reassigned to HIP Basic and HIP State Basic tiers. Additional HIP plan details may be found in the Indiana Health Coverage Programs Policy Manual and Indiana Health Coverage Programs Provider Reference [23]. Abbreviations: PHE, Public Health Emergency; HIP, Healthy Indiana Plan; FPL, Federal Poverty Level; POWER, Personal Wellness and Responsibility; ED, emergency department.

We analyzed restricted‐access data from the Indiana Family and Social Services Administration. This study was deemed exempt by Indiana University's Institutional Review Board in accordance with 45 CFR 164.512(i). Using individual‐level Indiana Medicaid enrollment data from January 2018 through December 2023, we constructed monthly enrollment counts for four plans: HIP Plus, HIP Basic, HIP State Plus, HIP State Basic. Figure 1 (on the left side) describes eligibility, benefits, and cost‐sharing structures of these four plans. We omitted the fifth main HIP plan, HIP Maternity, because enrollment in this plan is determined by pregnancy and not personal responsibility incentives. We excluded HIP Emergency Services Only (ESO) because it is not a HIP benefits plan. HIP ESO reimburses hospitals for costs incurred in providing critical medical and pregnancy care for people who are not eligible for Medicaid due to immigration status. We used self‐reported race to obtain monthly enrollment counts for members who are non‐Hispanic Black and non‐Hispanic White (ethnicity and race are collected on the application for Medicaid; recipients can select Hispanic or Latino ethnicity and denote which of the following race categories they identify as: Asian, Black or African American, White, American Indian or Alaska Native, Native Hawaiian or Pacific Islander, Multiracial). We opt to focus on the non‐Hispanic population because the rules around eligibility of Hispanic recipients are further complicated by their legal status in the U.Next, we calculated race‐specific rates of enrollment (in each of the four HIP plans) per 100 state residents, using race‐specific state population data from the US Census [22]. Then for the HIP plans and also for the HIP State plans, we calculated the proportion of recipients in the Plus plan relative to the total enrollment in both Plus and Basic plans, respectively, by race. Finally, we calculated disparity ratios, a ratio of the proportion of non‐Hispanic Black versus non‐Hispanic White enrollment in Plus plans. We are particularly interested in whether the disparity ratio approaches one indicating greater parity in non‐Hispanic Black and non‐Hispanic White members' enrollment in more comprehensive plans.

We used an interrupted time series design considering three policy changes. First, (A) Indiana suspended eligibility redeterminations and cost‐sharing in April 2020, locking new members into continuous coverage on HIP Plus. Members already enrolled in HIP Basic continued on HIP Basic with reduced benefits. Second, (B) Indiana's Medicaid office administratively shifted all members remaining on HIP Basic to the HIP Plus plan during July 2021. Despite having the annual opportunity to upgrade themselves to HIP Plus, now without monthly premiums, these members had stayed in HIP Basic with reduced benefits. In contrast, HIP State Basic members were not upgraded to HIP State Plus because both HIP State plans have the same enhanced benefits package, once their different cost‐sharing requirements were suspended. Third, (C) redeterminations resumed in April 2023. We analyze four policy intervals: the Pre‐PHE period from January 2018–March 2020 (“Pre‐PHE”), PHE period from April 2020–July 2021 (“Early PHE”), HIP Basic/Plus Upgrade period from August 2021–April 2023 (“HIP Plus Upgrade”), and Redetermination period from May 2023–December 2023 (“Redetermination”). Figure 1 (on the right side) marks the periods during which specific requirements were suspended and state administrative action occurred.

We estimated linear regression models including monthly linear time trends for each period (“period trend”), capturing the months since the start of each period, an indicator that takes a value of one following the start of each period (“post‐period indicator”), and the interaction of the post‐period indicator with the monthly linear period trend. A statistically significant coefficient on the post‐period indicator indicates immediate shifts in enrollment outcomes. Statistically significant coefficient estimates on the interaction of the post‐period indicator with the monthly linear period trend indicate changes in enrollment relative to the previous period. All models were estimated using 72 study months (i.e., 1/2018–12/2023), and regression‐adjusted monthly enrollment outcomes for members who are non‐Hispanic Black or non‐Hispanic White, stratified by HIP plan type, were presented graphically across the four policy environments during our study period. All models were estimated using heteroskedasticity and autocorrelation consistent (Newey–West) standard errors to account for serial correlation in monthly outcomes. As a sensitivity analysis, we estimated alternative specifications allowing for nonlinear time trends using natural cubic splines and compared predicted trajectories across models. We also examined unadjusted trends in enrollment outcomes for the full (combined non‐Hispanic Black and non‐Hispanic White) sample and separately in stratified subsamples of members who are non‐Hispanic Black or non‐Hispanic White.

## 3 Results

**FIGURE 2:**
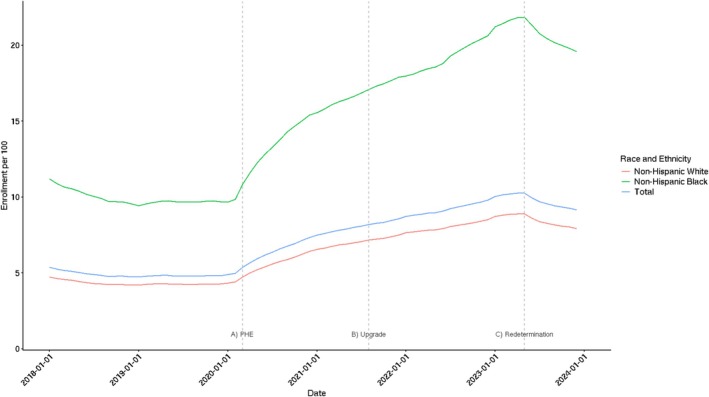
Trends in Non‐Hispanic Black and Non‐Hispanic White Enrollees Per 100 Residents in Healthy Indiana Plans. *Note:* Plot of total enrollment in both HIP plans and HIP State plans for non‐Hispanic White and non‐Hispanic Black enrollees. Enrollment is adjusted for total Indiana population by race, per month as estimated by the American Community Survey (ACS). “Total” enrollment is calculated as non‐Hispanic White + non‐Hispanic Black HIP and HIP State enrollees/non‐Hispanic White + non‐Hispanic Black state population estimate, per month. Vertical, dotted, gray lines indicate the time periods used to construct the ITS analysis. Red denotes non‐Hispanic White enrollees; green denotes non‐Hispanic Black enrollees, and blue denotes the total of non‐Hispanic White and non‐Hispanic Black enrollees. Abbreviations: HIP, Healthy Indiana Plan; ITS, interrupted time series; PHE, Public Health Emergency.

We identified 939,667 HIP members who self‐identified as non‐Hispanic White or non‐Hispanic Black (77.3% non‐Hispanic White, 22.7% non‐Hispanic Black) during the period 2018–23 (out of 1,233,520 people of any race enrolled in the four HIP plans of interest plus HIP Maternity). The state‐population‐adjusted rate of total non‐Hispanic White and non‐Hispanic Black HIP enrollees increased significantly from 4.89 to 10.27 per 100 residents between January 2020 and April 2023, an increase of 110.0% over this period (see Figure 2). By December 2023, the end of our study period, enrollment decreased to 9.16 per 100 residents, reflecting a decrease of 10.8% since the start of Indiana's Medicaid unwinding in May 2023.

Notably, the rates of enrollment differed by members who are non‐Hispanic White and non‐Hispanic Black over time. From January 2020 to April 2023, HIP rates increased from 4.34 to 8.90 per 100 residents for non‐Hispanic White members, an increase of 105.1%. HIP rates for non‐Hispanic Black members increased more quickly, from 9.68 to 21.82 per 100 residents, an increase of 125.4%. By December 2023, non‐Hispanic White HIP enrollment decreased to 7.93 per 100 residents, marking a decrease of 10.9%. Non‐Hispanic Black HIP enrollment decreased to 19.59 per 100 residents, a decrease of 10.2%.

**FIGURE 3:**
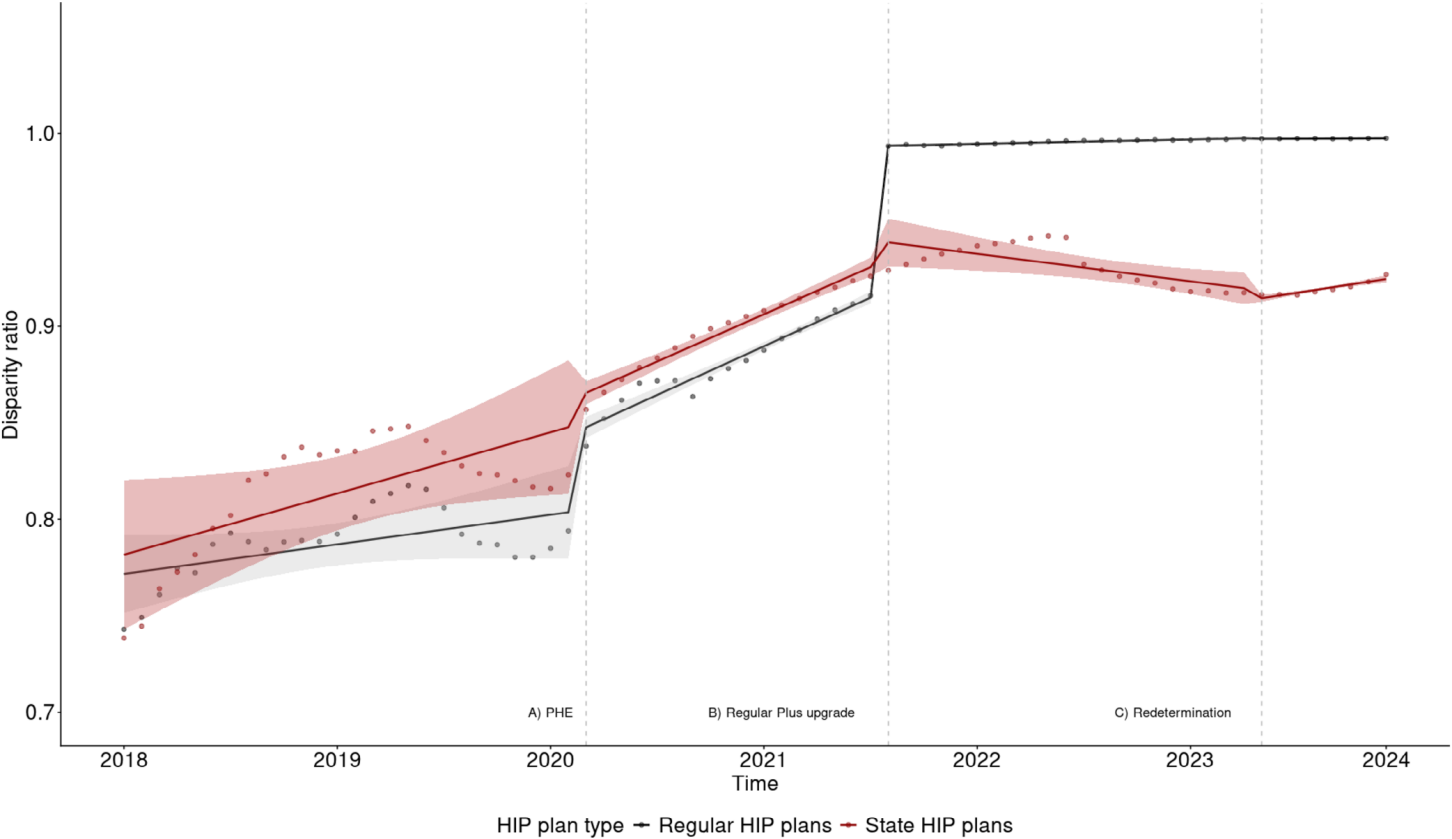
Disparity Ratios for HIP Plans and HIP State Plans for Non‐Hispanic Black and Non‐Hispanic White Enrollees. *Note:* Interrupted time series (ITS) plot of disparity ratios (proportion of non‐Hispanic Black and non‐Hispanic White Plus plan enrollment) for HIP plans (black, solid line) and HIP State plans (red, dotted line). Dots show the disparity ratio for each plan type calculated at each month, while lines show the estimated linear trend at each time period (with shaded regions showing the 95% confidence interval of those estimates). Vertical, dotted, gray lines indicate the time periods used to construct the ITS analysis. The disparity ratio represents the proportion of non‐Hispanic Black to non‐Hispanic White members' enrollment in more comprehensive (Plus) plans, with values closer to one signifying greater enrollment parity. Spline‐based sensitivity estimates are shown in Appendix A, Table S1. Abbreviations: HIP, Healthy Indiana Plan; ITS, interrupted time series; PHE, Public Health Emergency.

Figure 3 shows statewide trends in disparity ratios between non‐Hispanic Black and non‐Hispanic White HIP members, separated by HIP plans (solid line) and HIP State plans (dotted line). Vertical lines marking the aforementioned policy shifts (A, B, and C) allow us to track changes in the disparity ratio by plan comprehensiveness across four periods. During the Early PHE period, when redetermination and cost‐sharing were suspended (A), and during the HIP Plus Upgrade period (B), the disparity ratio decreased for those on the HIP plans. This shift indicates a decline in the disparity between enrollment of members who are non‐Hispanic Black and non‐Hispanic White in HIP Plus versus Basic plans, which is especially evident during the Upgrade period (B). The disparity ratio for members in HIP State plans increased following a similar trend as the HIP plans, but to a lesser extent. In sensitivity analyses allowing for nonlinear time trends, spline‐based predictions closely tracked the primary model estimates, with modest improvements in fit around transition periods but no change in the overall pattern of results (Appendix A, Figure S1).

In Table 1, we show changes in the disparity ratios across the four policy periods, using regression‐adjusted intercept and slope estimates with 95% confidence intervals (CIs) that account for serial correlation in monthly outcomes from our interrupted time series analysis. We focus our analysis on changes to the disparity ratio for HIP plans. We conclude the Results with a comparison to changes in the disparity ratio for HIP State plans. Results from alternative specifications allowing nonlinear time trends were substantively similar to those from the primary segmented linear models (Appendix A, Table S1).

**TABLE 1:** Interrupted time series regression results of disparity ratio.

	Pre‐PHE (*N* = 27 months)		Early PHE (*N* = 16 months)		HIP Plus Upgrade (*N* = 21 months)		Redetermination (*N* = 8 months)	
	Intercept (95% CI)	Slope (95% CI)	Intercept (95% CI)	Slope (95% CI)	Intercept (95% CI)	Slope (95% CI)	Intercept (95% CI)	Slope (95% CI)
HIP Plans	0.772*** (0.751, 0.792)	0.001 (0.000, 0.003)	(0.848)*** (0.842, 0.853)	0.004*** (0.004, 0.005)	0.994*** (0.993, 0.994)	0.000*** (0.000, 0.000)	0.997*** (0.997, 0.997)	0.000*** (0.000, 0.000)
Change from Previous Period			0.076*** (0.054, 0.097)	0.003*** (0.001, 0.005)	0.146*** (0.141, 0.151)	−0.004*** (−0.004, −0.004)	0.004*** (0.003, 0.004)	0.000*** (0.000, 0.000)
HIP State Plans	0.782*** (0.742, 0.821)	0.003** (0.000, 0.005)	0.866*** (0.859, 0.872)	0.004*** (0.003, 0.005)	0.943*** (0.931, 0.955)	−0.001** (−0.002, 0.000)	0.915*** (0.913, 0.916)	0.001*** (0.001, 0.002)
Change from Previous Period			0.084*** (0.044, 0.124)	0.001 (−0.001, 0.004)	0.077*** (0.063, 0.093)	−0.005*** (−0.006, −0.004)	−0.028*** (−0.041, −0.017)	0.003*** (0.002, 0.004)

*Note: p* < 0.10 = *, *p* < 0.05 = **, *p* < 0.001 = ***. Estimates are reported with 95% confidence intervals based on heteroskedasticity and autocorrelation consistent (Newey West) standard errors. The disparity ratio represents the proportion of non‐Hispanic Black to non‐Hispanic White members' enrollment in more comprehensive (Plus) plans, with values closer to one signifying greater enrollment parity.

Abbreviations: CI, confidence interval; HIP, Healthy Indiana Plan; PHE, Public Health Emergency.

Starting in January 2018, the disparity ratio for members who are non‐Hispanic Black versus non‐Hispanic White enrolled in the more comprehensive HIP Plus plan was 0.772 (95% CI, 0.751–0.792; *p* < 0.001). This indicates that HIP members who are non‐Hispanic Black were approximately 23 percentage points less likely to be enrolled in the more comprehensive HIP Plus plan compared to their non‐Hispanic White counterparts before the start of the PHE.

The early PHE period shows statistically significant increases in the disparity ratio, implying disparity reductions for HIP plans once premium payments, Basic downgrades, coverage terminations, and eligibility redeterminations were suspended. At the onset of the suspension (A), the disparity ratio increased by 0.076 points (95% CI, 0.054–0.097; *p* < 0.001) as compared to the previous period for HIP members. Over the course of the early PHE period, we observed an additional disparity ratio increase of 0.004 (95% CI, 0.004–0.005; *p* < 0.001) points per month for HIP members. Thus, following Indiana's suspension of cost‐sharing and redetermination requirements, the disparity between HIP members who are non‐Hispanic Black and non‐Hispanic White enrolled in more comprehensive plans decreased.

The HIP Plus Upgrade period remediated the outstanding disparity in HIP enrollment and plan comprehensiveness among non‐Hispanic Black and non‐Hispanic White members. At the onset of this period, the disparity ratio for HIP members had increased by 0.146 points (95% CI, 0.141–0.151; *p* < 0.001), to 0.994 (95% CI, 0.993–0.994; *p* < 0.001). Immediately following the HIP Plus upgrade, the previously increasing trend in the disparity ratio flattened (change: 0.000; 95% CI, 0.000–0.000; *p* < 0.001). Overall, the 23% pre‐PHE disparity between non‐Hispanic Black and non‐Hispanic White HIP Plus enrollment was almost completely reduced after Indiana's policy to upgrade HIP Basic members.

Finally, although Medicaid redetermination (C) restarted, Indiana continued its suspension of cost‐sharing throughout the time of our study. We did not observe any significant level changes for HIP members compared to the prior period. The disparity ratio remained at 0.997 (95% CI, 0.997–0.997; *p* < 0.001), and parity between non‐Hispanic Black and non‐Hispanic White enrollees continued from May 2023 to December 2023 (0.000; 95% CI, 0.000–0.000; *p* < 0.001). During the early months of the redetermination period, racial disparities in access to comprehensive coverage did not worsen as no one could be downgraded to HIP Basic for missed POWER Account payments.

Turning to the disparity ratio for members in HIP State plans (comprised of medically complex members), we observed a similar trend as the HIP plans, but to a lesser extent. After a similar increase in both disparity ratios during the Early PHE period, the HIP Plus Upgrade period indicates the differential effect on disparity ratios of the state's administrative upgrade (B) of HIP Basic members to Plus, while HIP State Basic members were not upgraded. Compared to the 0.146 points increase in the disparity ratio for HIP plans, a smaller, but still statistically significant increase of 0.077 points (95% CI, 0.063–0.093; *p* < 0.001) to 0.943 (95% CI, 0.931–0.955; *p* < 0.001) occurred in the HIP State plan disparity ratio during the HIP Plus Upgrade period. In addition, we did detect a statistically significant decrease of 0.028 points (95% CI, −0.041 to −0.017; *p* < 0.001) in the disparity ratio of HIP State members at the start of the Medicaid redetermination period, which decreased the disparity ratio to 0.915 (95% CI, 0.913–0.916; *p* < 0.001). At the end of the study, among those in HIP State plans, we continue to observe a persistent 8.5 percentage point gap in non‐Hispanic Black members' access to the more comprehensive State Plus plan relative to their non‐Hispanic White counterparts.

## 4 Discussion

Federal and state policies to suspend Medicaid eligibility redetermination during the PHE temporarily eased Medicaid administrative burdens nationwide [24, 25]. HIP members in Indiana also experienced a suspension of monthly premiums that paused a cascading series of personal responsibility incentives in the form of either coverage downgrades and copayments or coverage terminations for missed premiums. With these combined incentives in place before the PHE, HIP members who are non‐Hispanic Black were significantly less likely to be enrolled in more comprehensive health plans relative to members who are non‐Hispanic White. Specifically, HIP members who are non‐Hispanic Black more often owed the copayments for all healthcare services and medications on HIP Basic and HIP State Basic plans and more often lost dental and vision coverage on HIP Basic than members who are non‐Hispanic White.

Following Indiana's pause of personal responsibility and redetermination requirements, these specific racial disparities in HIP enrollment and plan comprehensiveness lessened but were not eliminated. Most HIP Basic members remained in their limited‐benefit plan despite having the annual opportunity to take personal action to upgrade their coverage to HIP Plus. More than a year into the PHE, the state's administrative action to reassign HIP Basic members to Plus removed this additional “sticky” racial disparity, resulting from members who are non‐Hispanic Black having been disproportionately downgraded to HIP Basic under HIP's prior rules.

Research finds that the costs of administrative burdens accumulate “over time” and that “the impact of these barriers is multiplied” when people experience a combination of administrative burdens [26, 27]. The persistence of members in the least comprehensive plan (HIP Basic), even after monthly premiums and Basic downgrades were suspended, indicates how HIP members can get stuck with benefit reductions over time. Once reassigned, HIP members face the consequence of added personal responsibility incentives on HIP Basic, likely increasing the difficulty of navigating each administrative burden; notably, the personal choice to upgrade to Plus. We find evidence that personal responsibility incentives may be particularly challenging for non‐Hispanic Black Medicaid enrollees, who are more likely to experience socioeconomic challenges and who have been more vulnerable to coverage downgrades in HIP [28, 29].

Scholars have highlighted Medicaid's role in advancing health equity, and this study finds evidence that administrative policies may both entrench and ameliorate inequities [30]. Indiana's policy change to upgrade HIP Basic members appeared to completely reduce the pre‐PHE HIP Plus enrollment disparity among members who are non‐Hispanic Black and members who are non‐Hispanic White, reversing prior patterns in which HIP Plus members who are non‐Hispanic Black were more likely to be downgraded to Basic. In contrast, the smaller improvement in the non‐Hispanic Black and non‐Hispanic White enrollment disparity in HIP State plans suggests that HIP's personal responsibility incentives are associated with racial disparity that persisted after the suspension of those incentives, in the absence of an administrative upgrade in State plans. This evidence underscores the importance of the state's proactive administrative upgrade policy, which did not extend to HIP State plans. Administrative policies impact minoritized groups differently, and policy actions at multiple levels (i.e., federal and state) targeting multiple aspects of Medicaid administration (e.g., cost‐sharing, eligibility determination, plan comprehensiveness, and waiver approval) may be needed to narrow disparities. Future research should continue to examine the consequences of Medicaid expansion for health equity with a particular focus on the role of administrative policies, such as personal responsibility provisions.

Administrative burdens are ubiquitous in health insurance, including Medicaid. Medicaid eligibility redetermination resumed in April 2023, and cost‐sharing requirements may resume in Indiana pending litigation [28]. This study finds that, in Indiana, racial disparity in plan comprehensiveness persists for members who are non‐Hispanic Black and non‐Hispanic White in the HIP State plans, which cover people with complex health conditions who tend to require more comprehensive care. If cost‐sharing returns, reduced coverage, in the form of routine copayments on HIP State Basic, will disproportionately affect non‐Hispanic Black members compared to non‐Hispanic White members. The co‐occurrence of higher rates of chronic health conditions, such as diabetes and hypertension, and lower SES among non‐Hispanic Black populations creates a “double disadvantage” for these members [31]. Our findings lend evidence that resuming Medicaid administrative burdens may exacerbate existing health inequities [32]. Further, if Indiana is allowed to reimplement premiums and the associated personal responsibility incentives, this change may place medically vulnerable non‐Hispanic Black populations at a heightened risk of inadequate access to care [6, 33].

Our analysis is not without limitations. Multiple concurrent changes during the PHE, including the suspension of eligibility redeterminations, the pause in cost‐sharing requirements, and broader labor market disruptions, limit the ability to attribute observed changes in disparity ratios to any single policy mechanism. Although recipients meet Medicaid eligibility criteria, the data do not capture variation in socioeconomic conditions within eligibility thresholds. As a result, observed differences by race may reflect both administrative factors and unobserved socioeconomic heterogeneity. Additionally, we focus on four HIP plans that have full benefits and personal responsibility incentives, which excludes recipients who were in HIP Maternity. Although our study population represents 76.18% of HIP members and ensures our study focuses on those whom the policy changes would affect the most, it limits our conclusions about recipients who are covered through HIP Maternity.

An additional consideration is that administrative data does not indicate reasons why HIP members were disenrolled or lacked coverage prior to the PHE. Higher rates of unemployment for non‐Hispanic Blacks due to the pandemic could bias our findings away from the null and are an alternative explanation to our findings. One of the benefits of examining recipients who qualify for the same plan is that they are in the same defined socioeconomic bracket to qualify; however, there is heterogeneity within this category in the amount and sources of wealth and employment experiences during the pandemic. Data showing significant reductions in disparities between non‐Hispanic Black and non‐Hispanic White enrollees continuing after the restart of redetermination, however, suggest that the suspended personal responsibility incentives, which remained paused at the end of this study, were a more decisive factor.

Moreover, because the disparity ratio reflects relative enrollment patterns across groups, reductions in disparities may arise from both a decline in the number of non‐Hispanic White members in the comprehensive plans and an increase in the number of non‐Hispanic Black members in the comprehensive plans. Due to similar enrollment trends for non‐Hispanic Black and non‐Hispanic White enrollees during the PHE, we can attribute movement toward parity as improvements for non‐Hispanic Black enrollees, rather than a decline in non‐Hispanic White enrollment in more comprehensive plans [34].

Furthermore, we lack a direct measure of administrative burdens experienced (i.e., costs incurred). Rather, we use the suspension of personal responsibility incentives and redetermination as a proxy for reduced administrative burden for all members. Although Indiana's combination of personal responsibility incentives is unique, HIP has served as a model for other states which include personal responsibility incentives, notably the POWER account. Our analysis may provide broader insights on the relationship between personal responsibility and Medicaid enrollment [32].

## 5 Conclusion

Personal responsibility provisions in Medicaid are associated with substantial and persistent differences in access to more comprehensive coverage. During the PHE, suspending premiums, copayments, and redeterminations—and notably suspending administrative downgrades into less comprehensive plans and coverage terminations—coincided with a narrowing of these gaps, while a discrete administrative upgrade largely eliminated differences in HIP Plus enrollment. In contrast, disparities persisted in HIP State plans, where comparable administrative action was not implemented.

These patterns point to the role of administrative design and cumulative burdens in shaping enrollment outcomes. Passive policy changes alone may not be sufficient to close gaps, whereas proactive administrative actions appear more effective. As states continue to design eligibility and cost‐sharing policies, these findings highlight the importance of policy design for maintaining equitable access to comprehensive coverage.

## Funding

This work was supported by Robert Wood Johnson Foundation.

## Conflicts of Interest

The authors declare no conflicts of interest.

## Supporting information


**Figure S1:** Disparity Ratios for HIP Plans and HIP State Plans for Non‐Hispanic Black and Non‐Hispanic White Enrollees with Spline Regression Overlay.
**Table S1:** Cubic Spline‐Based Interrupted Time Series Regression Results of Disparity Ratio.

## Data Availability

Research data are not shared.
