# Supplementary material for: Racial Disparities and Personal Responsibility Incentives in Medicaid
Source: Health Serv Res. 2026 Jun 10;61(3):e70139. doi: 10.1111/1475-6773.70139 (PMC13253046; doi:10.1111/1475-6773.70139)
Supplement: Supplementary file 1 — Figure S1: Disparity Ratios for HIP Plans and HIP State Plans for Non‐Hispanic Black and Non‐Hispanic White Enrollees with Spline Regression Overlay. Table S1: Cubic Spline‐Based Interrupted Time Series Regression Results of Disparity Ratio. [file HESR-61-e70139-s001.docx]

**Appendix A: Sensitivity Analysis**

**Figure A1. Disparity Ratios for HIP Plans and HIP State Plans for Non-Hispanic Black and Non-Hispanic White Enrollees with Spline Regression Overlay**


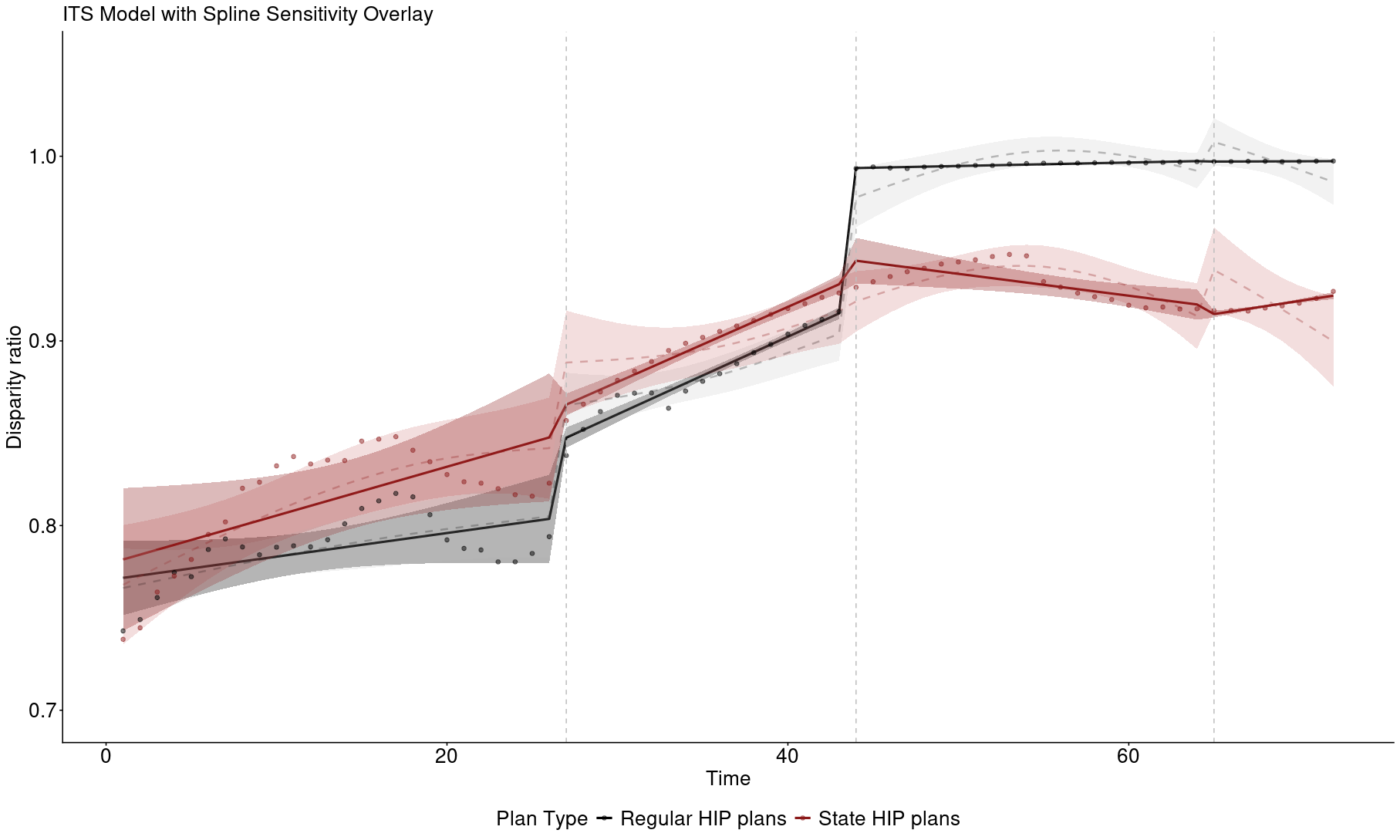


Note: Interrupted time series (ITS) plot of disparity ratios (proportion of non-Hispanic Black and non-Hispanic White Plus plan enrollment) for HIP plans (black, solid line) and HIP State plans (red, dotted line), HIP plans using the alternative cubic spline model specification (gray, dashed line), and HIP State plans using the alternative cubic spline model specification (light red, dashed line). Dots show the disparity ratio for each plan type calculated at each month, while lines show the estimated linear trend at each time period (with shaded regions showing the 95% confidence interval of those estimates). Vertical, dotted, grey lines indicate the time periods used to construct the ITS analysis. The disparity ratio represents the proportion of non-Hispanic Black to non-Hispanic White members’ enrollment in more comprehensive (Plus) plans, with values closer to one signifying greater enrollment parity.

**Table A1. Cubic Spline-Based Interrupted Time Series Regression Results of Disparity Ratio**

|  | Pre-PHE (N = 27 months) | | Early PHE (N = 16 months) | | HIP Plus Upgrade (N = 21 months) | | Redetermination (N = 8 months) | |
| --- | --- | --- | --- | --- | --- | --- | --- | --- |
|  | Intercept (95% CI) | Slope (95% CI) | Intercept (95% CI) | Slope (95% CI) | Intercept (95% CI) | Slope (95% CI) | Intercept (95% CI) | Slope (95% CI) |
| HIP Plans | 0.766*** (0.745, 0.788) | 0.002** (0.000, 0.003) | 0.865*** (0.848, 0.883) | 0.002** (0.001, 0.004) | 0.978*** (0.962, 0.994) | 0.001 (0.000, 0.002) | 0.978*** (0.962, 0.994) | -0.003** (-0.006, 0.000) |
| Change from Previous Period |  |  | 0.099*** (0.066, 0.132) | 0.001 (-0.001, 0.003) | 0.112*** (0.087, 0.138) | -0.002* (-0.004, 0.000) | 0.030** (0.007, 0.053) | -0.004** (-0.007, -0.001) |
| HIP State Plans | 0.768*** (0.736, 0.800) | 0.003** (0.001, 0.005) | 0.888*** (0.860, 0.916) | 0.002 (-0.001, 0.004) | 0.921*** (0.905, 0.938) | 0.000 (-0.001, 0.001) | 0.938*** (0.915, 0.961) | -0.006** (-0.010, -0.001) |
| Change from Previous Period |  |  | 0.120*** (0.072, 0.169) | -0.001 (-0.004, 0.002) | 0.033* (0.000, 0.067) | -0.002 (-0.005, 0.000) | 0.017 (-0.016, 0.050) | -0.005** (-0.010, 0.000) |

Note: p < 0.10 = *, p < 0.05 = **, p < 0.001 = ***. This model specification used cubic splines to address potential non-linear trends and seasonality. Reported regression results used heteroskedasticity- and autocorrelation-consistent (Newey-West) standard errors, which are robust to misspecification of the error structure. Period estimates are model-based predicted values at the beginning of each period. Slopes represent the average monthly change within each period, calculated as the difference between predicted values at the end and start of the period divided by the number of months in that period. Changes from previous periods were calculated as differences in predicted values (for levels) or average slopes between adjacent periods. The disparity ratio represents the proportion of non-Hispanic Black to non-Hispanic White members’ enrollment in more comprehensive (Plus) plans, with values closer to one signifying greater enrollment parity. Abbreviations: PHE, Public Health Emergency; CI, confidence interval; HIP, Healthy Indiana Plan.
